# Preventing White Adipocyte Browning during Differentiation *In Vitro*: The Effect of Differentiation Protocols on Metabolic and Mitochondrial Phenotypes

**DOI:** 10.1155/2022/3308194

**Published:** 2022-04-05

**Authors:** Elena Herbers, Mimmi Patrikoski, Anita Wagner, Riikka Jokinen, Antti Hassinen, Sini Heinonen, Susanna Miettinen, Hilkka Peltoniemi, Eija Pirinen, Kirsi H. Pietiläinen

**Affiliations:** ^1^Obesity Research Unit, Research Program for Clinical and Molecular Metabolism, Faculty of Medicine, University of Helsinki, Helsinki, Finland; ^2^Research Program for Clinical and Molecular Metabolism, Faculty of Medicine, University of Helsinki, Helsinki, Finland; ^3^Research Unit for Internal Medicine, Faculty of Medicine, University of Oulu, FIN-90220 Oulu, Finland; ^4^Institute for Molecular Medicine Finland, FIMM, University of Helsinki, Helsinki, Finland; ^5^Adult Stem Cells Group, Faculty of Medicine and Health Technologies, Tampere University, Tampere, Finland; ^6^Research, Development and Innovation Center, Tampere University Hospital, Tampere, Finland; ^7^Tilkka Hospital, Helsinki, Finland; ^8^Obesity Center, Abdominal Center, Helsinki University Hospital and University of Helsinki, Helsinki, Finland

## Abstract

Mitochondrial dysfunction in white adipose tissue is strongly associated with obesity and its metabolic complications, which are important health challenges worldwide. Human adipose-derived stromal/stem cells (hASCs) are a promising tool to investigate the underlying mechanisms of such mitochondrial dysfunction and to subsequently provide knowledge for the development of treatments for obesity-related pathologies. A substantial obstacle in using hASCs is that the key compounds for adipogenic differentiation *in vitro* increase mitochondrial uncoupling, biogenesis, and activity, which are the signature features of brown adipocytes, thus altering the white adipocyte phenotype towards brown-like cells. Additionally, commonly used protocols for hASC adipogenic differentiation exhibit high variation in their composition of media, and a systematic comparison of their effect on mitochondria is missing. Here, we compared the five widely used adipogenic differentiation protocols for their effect on metabolic and mitochondrial phenotypes to identify a protocol that enables *in vitro* differentiation of white adipocytes and can more faithfully recapitulate the white adipocyte phenotype observed in human adipose tissue. We developed a workflow that included functional assays and morphological analysis of mitochondria and lipid droplets. We observed that triiodothyronine- or indomethacin-containing media and commercially available adipogenic media induced browning during *in vitro* differentiation of white adipocytes. However, the differentiation protocol containing 1 *μ*M of the peroxisome proliferator-activated receptor gamma (PPAR*γ*) agonist rosiglitazone prevented the browning effect and would be proposed for adipogenic differentiation protocol for hASCs to induce a white adipocyte phenotype. Preserving the white adipocyte phenotype *in vitro* is a crucial step for the study of obesity and associated metabolic diseases, adipose tissue pathologies, such as lipodystrophies, possible therapeutic compounds, and basic adipose tissue physiology.

## 1. Introduction

Increasing rates of obesity and its associated metabolic comorbidities are important challenges for healthcare systems and have considerable social and economic impacts [1, 2]. The exact mechanisms by which obesity leads to impaired metabolic health are still poorly understood, but abnormal function of white adipose tissue (WAT) is considered to be a major driver of these changes [3–5]. Importantly, a growing body of evidence suggests that mitochondrial dysfunction is associated with an impaired WAT phenotype in obesity [6–9].

Mitochondria are subcellular organelles that produce aerobic energy through oxidative phosphorylation (OXPHOS). Mitochondria are crucial for adipocyte differentiation (adipogenesis) and for the main adipocyte functions, such as oxidation of fatty acids (FA), glucose, and branched-chain amino acid (BCAAs) [10–16]. The fact that these processes are downregulated in WAT in obesity and associated with mitochondrial dysfunction highlights the important role of mitochondria in adipose tissue [17–25]. Recent animal studies have shown that mitochondrial activation in WAT can be potentially used as a therapeutic strategy for obesity and metabolic disorders [26]. Activating WAT mitochondria can lead to browning of white adipocytes (i.e., acquisition of brown adipocyte features). Brown adipocytes are characterized by a dense mitochondrial network, increased mitochondrial number and respiration, uncoupling 1- (UCP1-) mediated thermogenesis, and elevated FA oxidation (FAO) [27–29]. The therapeutic effect of white adipocyte browning is based on increased oxidative and uncoupling capacity of mitochondria. However, only few studies have been conducted on mitochondrial activation in white adipocytes in humans, and further investigations are necessary.

The current bottleneck in the development of WAT mitochondrial activating compounds is the lack of proper white adipocyte *in vitro* models. Mouse 3T3-L1 cells, human Simpson-Golabi-Behmel syndrome (SGBS) preadipocytes, and primary stromal/stem cells derived from umbilical cord blood, bone marrow, or adipose tissue are currently the most extensively used *in vitro* models [30, 31]. Human primary adipose stromal/stem cells (hASCs) are easily accessible, capable of adipogenic differentiation *in vitro*, preserve donor- and depot-specific differences, and therefore are a promising tool for obesity research [32, 33]. However, the great diversity of hASC adipogenic differentiation protocols, including varying components and their concentrations and induction time, significantly limits the utilization and reproducibility of hASCs as an *in vitro* model (Supplementary Table 1). A substantial challenge is that some of the common compounds in adipogenic differentiation media, such as PPAR*γ* agonists and triiodothyronine (T3), can induce browning of *in vitro*-differentiated white adipocytes by boosting mitochondrial uncoupling, biogenesis, and activity and subsequently alter the metabolic profile of the cells [9, 34–40]. Minimizing browning and preserving the original adipocyte phenotype are essential when studying white adipocyte biology and mitochondrial function. To date, the mechanisms behind the browning effect of adipogenic compounds remain to be elucidated, and a direct comparison of the various differentiation protocols on mitochondrial and adipocyte phenotypes has not been performed.

In this study, we investigated the effect of the commonly used white adipocyte differentiation protocols on the white adipocyte metabolic and mitochondrial phenotype. We used a robust workflow to evaluate lipid and mitochondrial parameters to identify the optimal *in vitro* differentiation conditions without excessive browning effect on the adipocytes. Our study revealed elevated browning markers in differentiation protocols where T3 or indomethacin (INDO) has been added to the media and also in the protocol where commercial media were used. In contrast, low doses and a short induction period with the PPAR*γ* agonist rosiglitazone (Rosi) ensured an efficient differentiation with a minimal browning effect. We suggest an optimal differentiation cocktail for human white adipocytes that limits browning and ensures a reliable utilization of hASCs in obesity research.

## 2. Materials and Methods

### 2.1. Reagents

All reagents used in cell culture and assays were obtained from Sigma-Aldrich (St. Louis, Missouri, USA) if not otherwise specified.

### 2.2. Characteristics of the WAT Sample Donors

Subcutaneous WAT samples were acquired from the abdominal area during an elective liposuction operation in Tilkka Hospital, Helsinki. The samples were obtained from 6 healthy female donors (age 38.6 ± 6.9 years) with BMI = 23.4 ± 2.6. All participants provided written informed consent. The study protocol was approved by the Ethics Committee of Helsinki University Hospital (HUS/1039/2019).

### 2.3. Isolation and Culture of hASCs

Subcutaneous WAT was immediately dissected from fibrous material and blood vessels after the surgery and transported to the laboratory. hASCs were extracted by using a mechanical and enzymatic isolation method as described earlier [41, 42]. Briefly, samples were minced into small fragments and digested in DMEM/F12 containing collagenase type 1 (1.5 mg/ml, Thermo Fisher Scientific) for 60-90 minutes with gentle shaking. The resulting cell suspension was centrifuged for 10 minutes at 600 × *g*, and the stromal vascular fraction was resuspended in growth medium containing 5% human serum (Biowest), 1% penicillin/streptomycin, and 1% GlutaMax (Thermo Fisher Scientific), seeded in tissue culture-treated T75 flasks, and cultured in the same medium at 37°C in 5% CO_2_. After 48 hours, the floating cells were washed out with PBS and growth medium was replaced. After reaching 80% confluence, cells were either passaged or frozen. The first passage after extraction was designated as passage 0, and cryopreserved cells in passages 2 to 5 were used in the experiments.

### 2.4. Adipogenic Differentiation of hASCs

The common differentiation conditions for white adipocytes are summarized in Supplementary Table 1. From these, we selected the five most regularly used protocols for our study (Table 1) with slight modifications to ensure uniform incubation times and basic compounds used (such as dexamethasone, IBMX, biotin, pantothenate, and serum type) between the protocols. We tested and compared protocols for their effects on mitochondrial and adipocyte-specific functions (Table 2). Adipogenic differentiation started on the second day of postconfluence (designated as Und), followed by an induction period (7 days) and maintenance period (14 days). Cells at day 21 of differentiation were used for experiments. For protocol 4, induction cocktail was used during the whole differentiation period as previously described [11, 43]. The maintenance medium was DMEM/F12 (Thermo Fisher Scientific) supplemented with 1% penicillin/streptomycin (Thermo Fisher Scientific), 1% GlutaMax (Thermo Fisher Scientific), 3% human serum (Biowest), 17 *μ*M pantothenate, 33 *μ*M biotin, 100 nM insulin, and 1 *μ*M dexamethasone to standardize the basic conditions. We replaced FBS with human serum (HS) in protocols 1-4 to avoid xenobiotic compounds. For the induction period, the maintenance media were supplemented as follows. Protocol 1 (P1) was based on Lee and Fried [44] and contained maintenance medium supplemented with 1 *μ*M Rosi (Calbiochem, San Diego, US), 2 nM T3, and 10 mg/ml transferrin. For protocol 2 (P2), T3 and transferrin were omitted. Protocol 3 (P3) was similar to P2, but Rosi concentration was decreased to 100 nM. Protocol 4 (P4) was based on Zhang et al. and supplemented with 850 nM insulin and 125 nM INDO [11]. For protocol 5 (P5), commercially available differentiation and maintenance media were used (DM-2 and AM-1, ZenBio, USA). This medium contained DMEM/F12, FBS, penicillin, streptomycin, amphotericin B, biotin, pantothenate, insulin, dexamethasone, IBMX, and PPAR*γ* agonist, although the exact concentrations of medium compounds are unknown.

### 2.5. Oil Red O Staining

Intracellular lipid accumulation was evaluated with Oil Red O (ORO) staining as described previously [45]. Briefly, cells were washed with PBS and fixed for 1 hour with 4% paraformaldehyde in PBS (Alfa Aesar). Subsequently, cells were pretreated with 60% isopropanol and incubated with 0.2% ORO staining solution for 15 minutes at room temperature. After a wash with PBS, ORO was extracted by a 10-minute incubation with 100% isopropanol. Absorbance was then measured at 510 nm with a Spark microplate reader (Tecan Group Ltd., Switzerland). Prior to assay measurement, cell nuclei were stained with Hoechst 33342 (1 : 1000) and imaged with a plate reader microscope Cytation5 (BioTek Instruments Inc., Vermont, U.S.). ORO assay results were normalized to cell number.

### 2.6. Gene Expression Analysis

Total RNA was extracted from the adipocytes of each of the protocols after *in vitro* differentiation for 21 days, from WAT, and from the mature adipocyte fraction (MAF) of the same subjects using the miRNeasy Mini Kit (QIAGEN, Venlo, Netherlands) according to the manufacturer's instructions. RNA concentration was assessed with a spectrometer (DeNovix), and quality was evaluated with TapeStation (Agilent, Santa Clara, USA). Total RNA (2 *μ*g) from each sample was reverse transcribed using the SuperScript VILO cDNA synthesis kit following the manufacturer's instructions (Invitrogen). The mRNA levels of nuclear-encoded (*ZIC1*, *TBX1*, *TMEM26*, *LEP*, *ADIPOQ*, *FABP4*, *PPARγ*, *PGC1α*, *PGC1β*, *UCP1*, *UCP2*, *CIDEA*, *LIPE*, *ACADM*, and *ACADS*) and mitochondrial-encoded (*MT-ND5*, *MT-COXI*) genes (Supplementary Table 2) were analyzed using quantitative PCR (qPCR) amplification in a CFX Real-Time system C1000Touch (Bio-Rad) with SYBR-green Flash (Thermo Fisher) with the following thermoprofile: 7 minutes at 95°C followed by 40 cycles of a 2-step amplification (denaturation at 95°C for 10 seconds and annealing and elongation at 60°C for 30 seconds). 40 ng of cDNA and 0.5 *μ*M primers were used for the reaction. Samples were measured in triplicate. The results were normalized to *IPO8* and *GUSB* (Supplementary Table 2) housekeeping genes, and relative gene expression levels were calculated with the standard curve method using the qBASE (Biogazelle) software [46]. Primer sequences are available in Supplementary Table 2.

### 2.7. Assessment of Mitochondrial DNA Amount

Total cellular DNA was isolated using proteinase K and SDS lysis followed by phenol : chloroform extraction and ethanol precipitation [47]. Total DNA was quantified using Qubit Fluorometer (Thermo Fisher). The amounts of DNA from mitochondrial (MT-ND5, MT-CYTB) and nuclear (B2M, APP) (Supplementary Table 2) genome regions were analyzed by qPCR amplification in CFX Real-Time system C1000Touch (Bio-Rad) with SYBR-green Flash (Thermo Fisher) with the following thermoprofile: 7 minutes at 95°C followed by 40 cycles of a 2-step amplification (denaturation at 95°C for 10 seconds and annealing and elongation at 60°C for 30 seconds). 10 and 1 ng of total DNA were used as a template for nuclear and mitochondrial genome region analyses, respectively, using 0.5 *μ*M primer concentration. Samples were measured in triplicate. The ratio between mitochondrial DNA (mtDNA) and nuclear DNA amount was used to determine the mtDNA amount with the standard curve method using the qBASE (Biogazelle) software [46]. Primer sequences are available in Supplementary Table 2.

### 2.8. Protein Extraction and Immunoblotting

Total protein from the cultured cells was extracted with RIPA lysis buffer (50 mM Tris HCl pH 7.6, 150 mM NaCl, 1 mM EDTA, 1% TX-100, 0.25% deoxycholic acid 97%, 0.5% SDS, and 1 mM PMSF in ETOH), and protein concentration was measured using the Pierce bicinchoninic acid (BCA) protein detection kit (Thermo Fisher Scientific, Rockford, IL, USA) according to the manufacturer's instructions. Protein quantities of OXPHOS complex subunits per mitochondria and per cell were measured via immunoblotting. The protein extracted from mouse brown adipose tissue (BAT) served as a control for UCP1. Proteins were reduced with 5% beta-mercaptoethanol in Laemmli buffer. Electrophoresis was performed with initial voltage 80 V and then 135 V after entering the separating gel. The protein was transferred to the nitrocellulose membrane and blocked with 5% skim milk-Tris-buffered saline-Tween 20 (TBST) for 1 hour at 4°C. The primary antibodies used were human OXPHOS cocktail (1 : 1000, OXPHOS cocktail Abcam, ab110411), vinculin (1 : 2500, Abcam ab129002), porin/VDAC1 (1 : 1000, Abcam ab15895), and UCP1 (1 : 1000, Novus Biologicals MAB6158). Primary antibodies were incubated overnight at 4°C. The secondary antibodies used were HRP-conjugated anti-rabbit and anti-mouse immunoglobulins (1 : 2500, Santa-Cruz) and were incubated for 2 hours. The protein band intensities were quantified using ImageLab software version 5.2.1 (Bio-Rad).

### 2.9. Immunocytochemistry

hASCs were seeded at a density of 10000 cells per well in a 96-well plate (CellCarrier-96 Ultra, PerkinElmer) and differentiated for 21 days. Cells were then fixed with 4% paraformaldehyde in PBS (Alfa Aesar) for 15 minutes. Lipid droplets and nuclei were stained with Bodipy green 488 0.5 *μ*g/ml and Hoechst 33342 1 : 1000 (Thermo Fisher Scientific, Waltham, Massachusetts, USA), respectively. Samples were analyzed with high-content screening microscopy using a PerkinElmer Opera Phenix spinning disk confocal microscope. The setup included a 63x water-immersion objective (NA 1.15, depth of focus 1.0 *μ*m) and two excitation lasers (405 nm with emission band-pass filter 435/480 and ex 488, em 500/550). On average 75 fields of view with 5% overlap were imaged per well using six predetermined *Z*-focus planes with 1 *μ*m plane distance and laser-based autofocusing. The images were captured with two Andor Zyla sCMOS cameras (16-bit, field of view 650 × 650 *μ*m^2^, effective xy resolution 0.66 *μ*m). The lipid droplet in the cells was classified and quantified with Harmony 4.9 software (PerkinElmer) with the parameters shown in Supplementary Table 3. In short, individual nuclei were segmented based on nuclear Hoechst staining, and the cytoplasmic region was determined accordingly. Secondly, the differentiated cell population was identified using a supervised machine learning classifier, which utilized the intensity and texture parameters of lipid droplets in the cell region (nucleus and cytoplasm) as visualized with the Bodipy (green) channel. Finally, lipid droplet parameters were assessed as separate segmented subpopulations in the Bodipy (green).

### 2.10. Transmission Electron Microscopy

Cells were differentiated on cover slips and fixed in 0.1 *μ*M sodium phosphate buffer pH 7.4 (A18139.14, VWR, Radnor, PA, USA) containing 2% glutaraldehyde for 1 hour at room temperature. Samples were processed following standard published methods and imaged with a Jeol JEM-1400 transmission electron microscope (TEM) (Jeol Ltd., Tokyo, Japan) operating at 80 kV [48]. Lipid droplets and mitochondrial parameters were analyzed using the Microscopy Image Browser (MIB) [49].

### 2.11. Mitochondrial Bioenergetic Profiling

Mitochondrial respiration was measured in differentiated adipocytes using the XF96e cellular flux analyzer (Seahorse Biosciences, North Billerica, MA, USA) using the Agilent Mito Stress Test. hASCs were seeded at a density 10000 cells per well and differentiated for 21 days. On the day of assay, maintenance medium was replaced with the Seahorse Assay medium supplemented with 10 mM glucose, 1 mM pyruvate, and 2 mM glutamine, and pH was adjusted to 7.4. Oxygen consumption rate (OCR) was measured at basal level and after addition of OXPHOS modulators, including proton leak after oligomycin (1 *μ*M), maximal OCR after FCCP (2 *μ*M), and nonmitochondrial respiration after antimycin A and rotenone (2 *μ*M each). All parameters were calculated as described previously [50]. After the measurement, cell nuclei were stained with Hoechst 33342 (1 : 1000) and imaged with a plate reader microscope Cytation5 (BioTek Instruments Inc., Vermont, U.S.). Results were normalized to cell number.

### 2.12. Lipolysis

Lipolysis experiments were performed at day 21 of differentiation as described previously [51]. Briefly, cells were washed with PBS and preincubated with basal DMEM without serum for 2 hours at 37°C for serum starvation. For stimulated and basal lipolysis measurement, adipocytes were then incubated for 2 hours at 37°C with Hank's Balanced Salt Solution (HBSS) containing 2% BSA (FA-free) with or without 1 *μ*M of isoproterenol, respectively. Conditioned media were collected, and free glycerol concentrations were quantified using a commercial fluorometric assay (EnzyChrome™ Adipolysis assay kit, BioAssay Systems, Hayward, CA, USA). Results were normalized to protein content of the cell lysate using a BCA kit.

### 2.13. Adipokine Secretion

Total adiponectin and leptin levels in the cell culture supernatants were quantified with the Human Total Adiponectin Quantikine ELISA kit and Human Leptin Quantikine ELISA kit (R&D Systems, Minneapolis, MN, USA). Conditioned media were collected between days 14 and 21 and centrifuged to discard cell debris. The samples were diluted for leptin measurement 1 : 40 and for adiponectin measurement 1 : 10. The assay was performed according to the manufacturer's instructions, and the absorbance was then measured at 450 nm with a Spark microplate reader (Tecan Group Ltd., Switzerland).

### 2.14. Statistics

Statistical analyses for the differences between the five protocols were performed by one-way ANOVA for multiple comparisons with the Tukey post hoc test using Prism GraphPad. Data are shown as mean with error bars representing SEM. Associations between the differentiation ratio and adipogenesis gene expression was performed using the Pearson correlation in Prism GraphPad.

## 3. Results

### 3.1. Comparison of Human White Adipocyte Differentiation Protocols In Vitro

This study included the five widely used adipocyte differentiation protocols, which were compared for their effects on adipocyte phenotype and mitochondrial uncoupling, biogenesis, morphology, and function (Table 2). As shown in Supplementary Table 1,1, the composition of differentiation media varied greatly between these commonly used protocols. We focused on comparing the following differentiation protocols: insulin 100 nM+Rosi 1 *μ*M+T3+transferrin (P1), insulin 100 nM+Rosi 1 *μ*M (P2), insulin 100 nM+Rosi 100 nM (P3), and insulin 850 nM+no Rosi+NDO (P4) (Table 1). In addition, we included one commonly used commercial differentiation medium (P5), which contains an unspecified PPAR*γ* agonist with unknown concentration as an inducer of differentiation. The concentrations of basic compounds, such as dexamethasone, IBMX, biotin, and pantothenate, were the same in all tested protocols, and their concentrations were based on the commonly used protocols (Supplementary Table 1).

### 3.2. Differentiation Capacity, Lipid Accumulation, and Lipid Droplet Parameters

**Figure 1:**
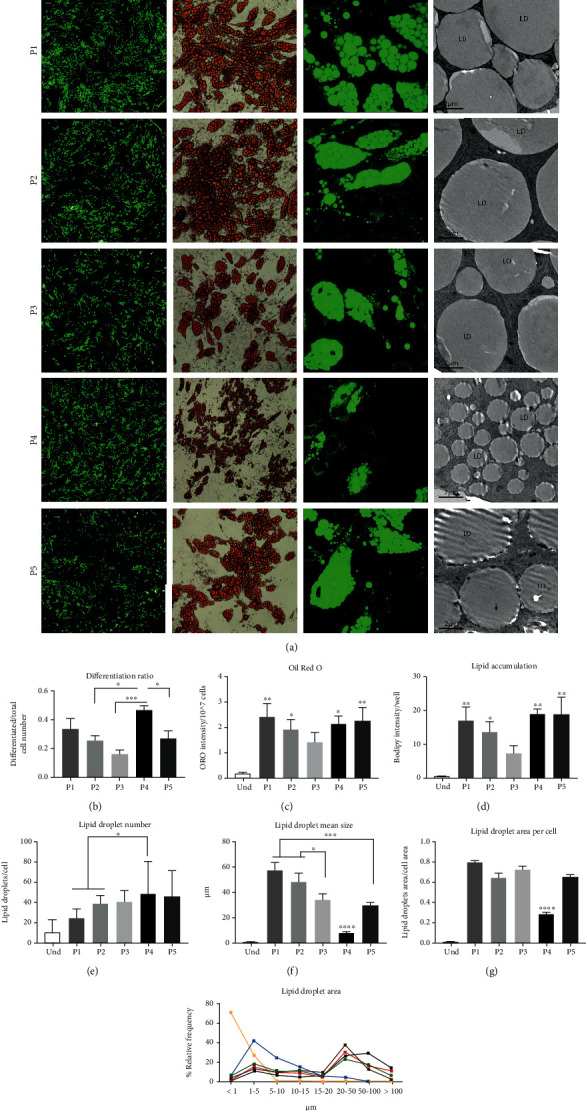
Effect of the differentiation protocols on differentiation capacity, lipid accumulation, and lipid droplet morphology. (a) Human adipose-derived stem cells were differentiated for 21 days with five adipogenic differentiation protocols. Cells were stained with Bodipy and imaged with 5x magnification for differentiation ratio calculation and with 63x magnification for lipid accumulation and lipid droplet morphology with a PerkinElmer Opera Phenix confocal microscope. Cells were stained with Oil Red O for lipid accumulation measurement and imaged at 10x magnification with a Nikon Eclipse TS100 brightfield microscope. Cells were imaged with a 1500x magnification JEOL JEM-1400 electron microscope for lipid droplet morphology. (b) The population of differentiated cells was calculated using Harmony software as a ratio of differentiated cells stained with Bodipy to total cell number based on nuclear staining with Hoechst 33342. (c) Oil Red O was eluted, absorbance was measured at 510 nm, and results were normalized to the cell number measured with Cytation5 after nuclear staining with Hoechst 33342. (d) Lipid accumulation per well was measured by quantification of Bodipy green intensity per cell population with Harmony software. Quantification of (e) lipid droplet number, (f) lipid droplet mean size, and (g) lipid droplet area per cell was imaged by transmission electron microscopy (TEM) and analyzed with the Microscopy Image Browser (MIB). (h) Relative frequencies of lipid droplet by size analyzed with MIB. hASC3 was used for TEM analysis. For each condition, at least 200 lipid droplets (80 lipid droplets for undifferentiated cells) were analyzed. Statistical analysis was performed using one-way ANOVA followed by Tukey's post hoc analysis (*n* = 6): ^∗^*p* < 0.05, ^∗∗^*p* < 0.01, ^∗∗∗^*p* < 0.001, and ^∗∗∗∗^*p* < 0.0001. Error bars are shown as SEM. The statistical differences in (c) and (d) indicate a comparison to undifferentiated hASCs, whereas that in (g) indicates a comparison with all the other protocols.

The main criteria for efficient adipogenic differentiation traditionally include good differentiation capacity, high lipid accumulation, and formation of large unilocular lipid droplets. Lipid droplet morphology is a distinct feature between white and brown adipocytes. White *in vitro*-differentiated adipocytes develop large unilocular lipid droplets that occupy nearly the whole cell area, whereas brown adipocytes form multilocular smaller droplets [27–29]. To test whether all protocols successfully induce adipogenic differentiation, we assessed the ratio of differentiated adipocytes to total cell number using immunocytochemistry (ICC) (Figures 1(a) and 1(b), Supplementary Figure 1(a)). The lowest differentiation ratio was observed in P3 (Figure 1(b)), suggesting that Rosi at concentration 100 *μ*M was not sufficient to stimulate appropriate differentiation, whereas the other protocols successfully differentiated hASCs into adipocytes. Lipid quantification with both ORO- and Bodipy-based methods showed a significant lipid accumulation compared to undifferentiated cells in all protocols except P3 (Figures 1(c) and 1(d)). We used TEM (Figure 1(a)) to further analyze lipid droplet parameters, including the number and size of lipid droplets per cell and the area that lipids occupied in the cell. While P4-differentiated adipocytes had the highest number of lipid droplets per cell (Figure 1(e)), the droplets were significantly smaller and showed a lower cell area occupied by the droplets compared with other protocols as measured by both ICC and TEM (Figures 1(f) and 1(g), Supplementary Figure 1(b)–(c)). The relative frequency of lipid droplet populations by area showed that most P4 droplets had an area distributed between 1 and 20 *μ*m without large droplets over 50 *μ*m. Although all adipocytes differentiated with the P1, P2, P3, and P5 protocols developed large lipid droplets, the P1 and P2 protocol adipocytes had the greatest proportion of droplets over 100 *μ*m (Figure 1(h)). Taken together, these results indicate that the INDO-based differentiation protocol (P4) induces brown-like multilocular white adipocytes, while P2 (Rosi) and P1 (Rosi+T3) protocols generate the whitest adipocytes based on lipid droplet parameters.

### 3.3. Gene Expression Profiles of Differentiated Adipocytes

**Figure 2:**
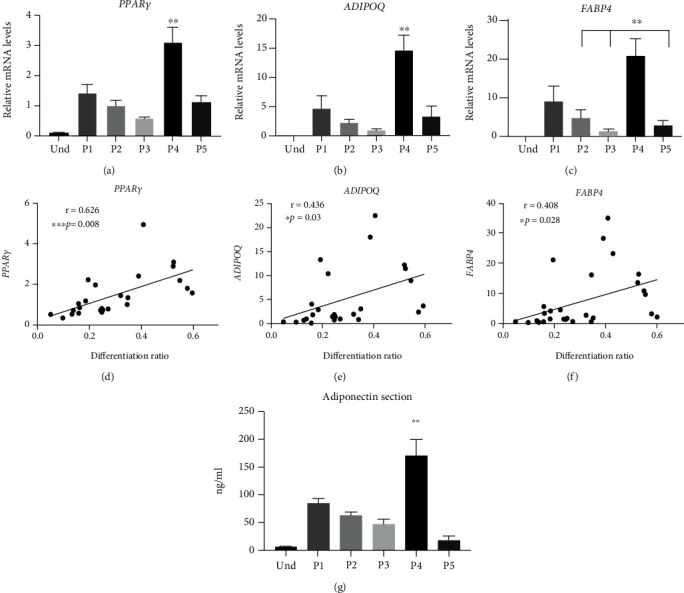
Effect of the differentiation protocols on adipogenesis gene expression. Human adipose-derived stem cells were differentiated for 21 days with five adipogenic differentiation protocols. Relative mRNA expression of nuclear-encoded genes (a) *PPARγ*, (b) *ADIPOQ*, and (c) *FABP4* was measured by RT-qPCR. Results were normalized to the expression of reference genes *IPO8* and *GUSB* using qBASE software. Statistical analysis was performed using one-way ANOVA followed by Tukey's post hoc analysis (*n* = 6): ^∗∗^*p* < 0.01. Error bars are shown as SEM. Expression correlation for relative mRNA expression of (d) *PPARγ*, (e) *ADIPOQ*, and (f) *FABP4* with the differentiation ratio. Statistical analysis was performed using the Pearson correlation (*n* = 6): ^∗^*p* < 0.05, ^∗∗∗^*p* < 0.001. (g) Secretion of adiponectin to the cell culture media. Statistical analysis was performed using one-way ANOVA followed by Tukey's post hoc analysis (*n* = 5): ^∗∗^*p* < 0.01. Error bars are shown as SEM. The statistical differences in (a), (b), and (g) indicate a comparison with all the other protocols.

**Figure 3:**
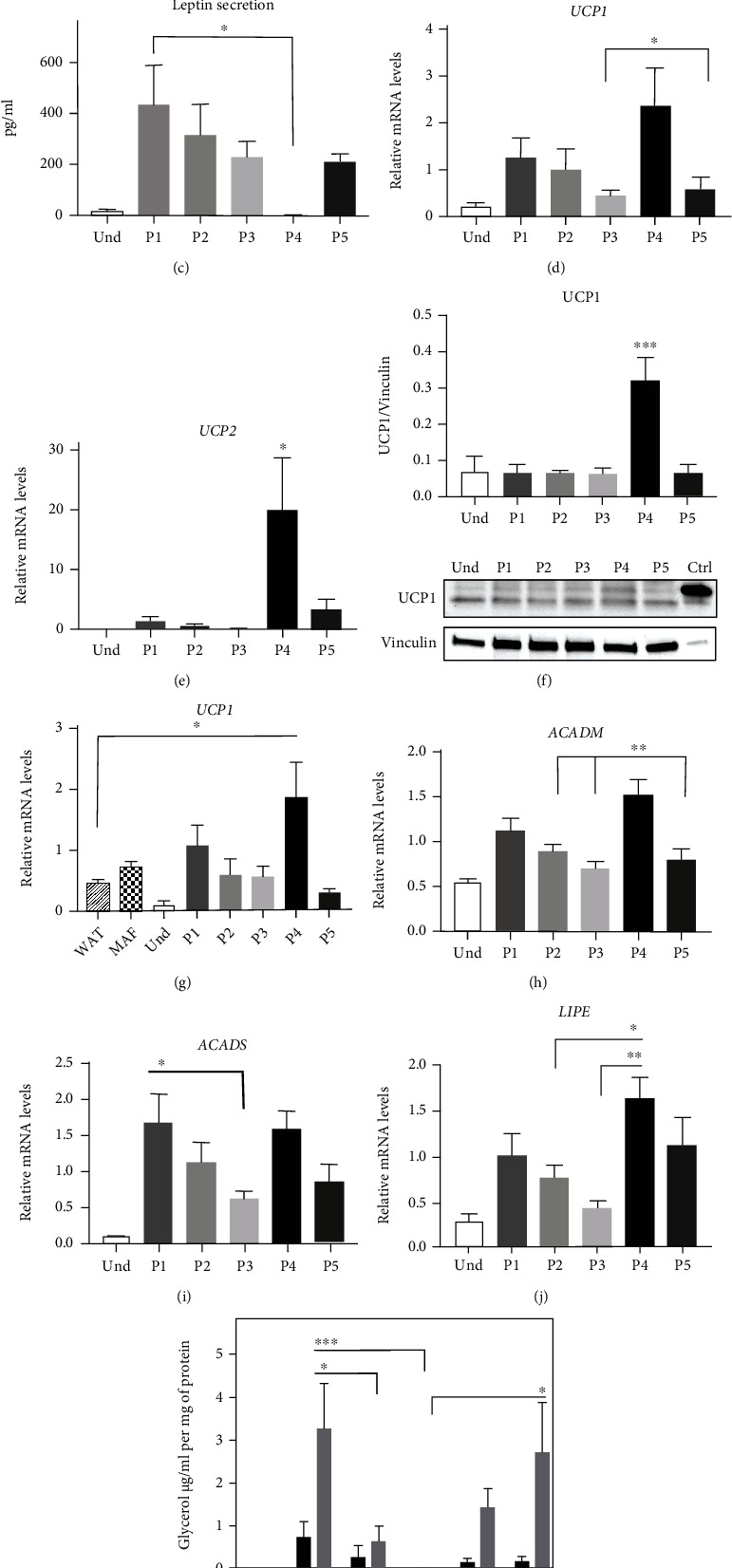
Effect of the differentiation protocols on white and brown adipocyte markers and lipid metabolism gene expression. Human adipose-derived stem cells were differentiated for 21 days with five adipogenic differentiation protocols. Relative mRNA expression of genes (a) *CIDEA*, (b) *LEPTIN*, (d) *UCP1*, and (e) *UCP2* measured by RT-qPCR. (c) Secretion of leptin to the cell culture media. (f) Protein levels of UCP1 and vinculin were analyzed by western blotting and quantification of UCP1 level relative to vinculin. (g) Relative mRNA expression of *UCP1* in *in vitro*-differentiated adipocytes, white adipose tissue (WAT), and mature adipocyte fraction (MAF) of the same subjects measured by RT-qPCR. Relative mRNA expression of genes (h) *ACADM*, (i) *ACADS*, and (j) *LIPE* measured by RT-qPCR. Gene expression results were normalized to the expression of reference genes *IPO8* and *GUSB* using qBASE software. (k) Basal and isoproterenol-stimulated free glycerol release in media. Statistical analysis was performed using one-way ANOVA followed by Tukey's post hoc analysis (*n* = 6): ^∗^*p* < 0.05, ^∗∗^*p* < 0.01, and ^∗∗∗^*p* < 0.001. Error bars are shown as SEM. The statistical differences in (a), (e), and (f) indicate a comparison with all the other protocols.

To confirm adipogenic differentiation on a gene expression level, we analyzed the expression of the key adipogenic markers *PPARγ*, *ADIPOQ*, and *FABP4* (Figures 2(a)–2(c)). The transcript levels demonstrated a moderate correlation with the differentiation ratio data (Figures 1(b) and Figures 2(d)–2(f), showing the highest expression of the adipogenic markers in P4 and the lowest in P3. We also measured the adiponectin secretion into cell culture medium, which confirmed the *ADIPOQ* expression data (Figure 2(g) (g)). Next, we asked which of the tested protocols is the most browning neutral. For this, we first analyzed the gene expression markers of brown adipocytes [38, 52–55]. We did not detect any expression of brown adipocyte markers *ZIC1*, *TBX1*, and *TMEM26* in cells differentiated with any of the protocols (data not shown). However, levels of the browning marker *CIDEA* were significantly increased while levels of the white adipocyte marker *LEPTIN* were decreased with protocol P4, indicating browning with this protocol (Figures 3(a) and 3(b)). Similarly, leptin secretion was reduced in P4 (Figure 3(c)). Compared with white adipocyte mitochondria, brown adipocyte mitochondria exhibit elevated thermogenesis and FAO [56]. Therefore, we next assessed mRNA levels of the main uncoupling proteins and FAO enzymes. Expression of uncoupling proteins *UCP1* and *UCP2* was significantly upregulated only with protocol P4 (Figures 3(d) and 3(d)). We also analyzed the UCP1 expression on protein level with immunoblotting and found it significantly increased with protocol P4 (Figure 3(f)), in line with gene expression results (Figure 3(d)). To confirm these results with *ex vivo* samples, we compared the expression levels of *UCP1* in *in vitro*-differentiated adipocytes to *ex vivo* subcutaneous WAT and MAF. Our findings confirmed that differentiation protocols P1-P3 and P5 did not induce *UCP1* expression, as P1-P3 and P5 adipocytes exhibited similar *UCP1* expression levels as subcutaneous WAT and MAF, in contrast to P4 adipocytes, where *UCP1* expression levels were significantly higher than those in *ex vivo* WAT (Figure 3(g)). The mRNA levels of the key FAO enzymes *ACADM* and *ACADS* were increased with protocols P4 and P1, respectively (Figures 3(h) and 3(i)), indicating elevated FAO with these protocols. FAO in brown adipocytes depends on the availability of free FAs, which are generated through lipolysis along with the release of glycerol [57]. Gene expression levels of *LIPE*, a key enzyme involved in lipolysis, exhibited the same tendency as FAO enzymes (Figure 3(j)). Additionally, we analyzed the release of free glycerol into cell culture medium as an indirect measurement of lipolysis [35, 57]. We observed a significant increase (up to 4-fold) in isoproterenol-stimulated glycerol release in P1 and P5 compared with P2 and P3 (Figure 3(k)), suggesting a browning feature in P1 and P5 conditions compared to P2 and P3. Taken together, these data indicate that based on gene expression signature, lipolysis, and FAO, Rosi-based P2 and P3 are the most browning neutral protocols. In contrast, the INDO-based P4 differentiation protocol stimulates the most brown adipocyte-like phenotype (gene expression, lipolysis, and FAO). Increased lipolysis and FAO are also characteristics for P1 (Rosi+T3) and P5 (commercial).

### 3.4. Mitochondrial Bioenergetic Profile

**Figure 4:**
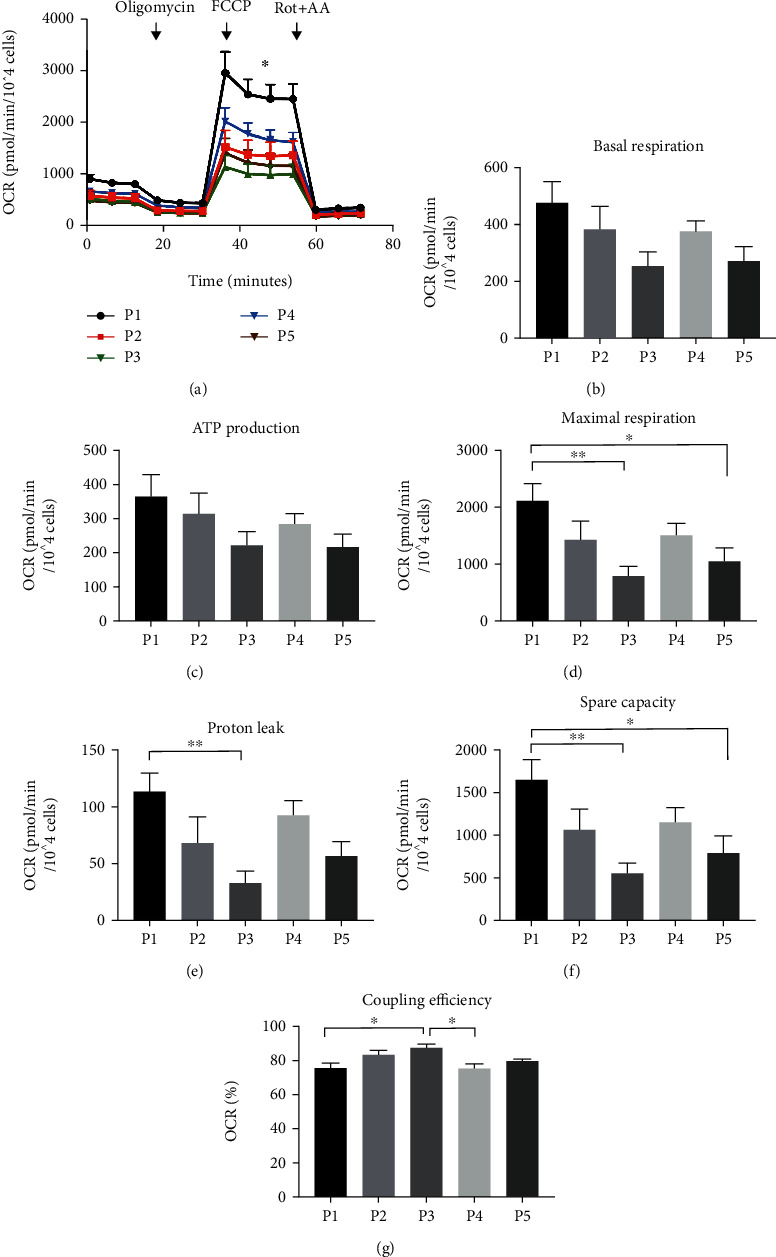
Effect of differentiation protocols on mitochondrial respiration. (a) Oxygen consumption rate (OCR) measured in adipocytes differentiated for 21 days with five differentiation media. Calculated average values of (b) basal respiration, (c) ATP production, (d) maximal respiration, (e) proton leak, (f) spare respiratory capacity, and (g) coupling efficiency (%) ((ATP production/basal respiration)∗100). Statistical analysis was performed using one-way ANOVA followed by Tukey's post hoc analysis (*n* = 6): ^∗^*p* < 0.05, ^∗∗^*p* < 0.01. Error bars are shown as SEM.

Since increased mitochondrial respiration and uncoupling are hallmarks of brown adipocytes [9, 58], we measured mitochondrial bioenergetic profiles with a Seahorse analyzer (Figure 4(a)). We did not detect any significant differences in basal respiration or ATP production between the protocols (Figures 4(b) and 4(c)). The protocol P3 and P5 adipocytes exhibited the lowest maximal respiration, proton leak (i.e., uncoupling), and spare respiratory capacity. In contrast, the P1 adipocytes had the highest maximal respiration, proton leak, and spare respiratory capacity (Figures 4(d)–4(f)) when compared with protocols P3 and P5. P2 and P4 adipocytes displayed a mitochondrial bioenergetic profile between the high and low groups. Moreover, coupling efficiency was lowest in P1 and P4 (Figure 4(g)), which suggests that the mitochondria of P1 and P4 adipocytes have low efficiency to couple electrons to ATP synthesis [50]. Overall, our data indicate that P2 (Rosi) induced a bioenergetic profile resembling that of white adipocytes, including moderate respiration level, proton leak, and high coupling efficiency.

### 3.5. Mitochondrial Biogenesis and Dynamics

**Figure 5:**
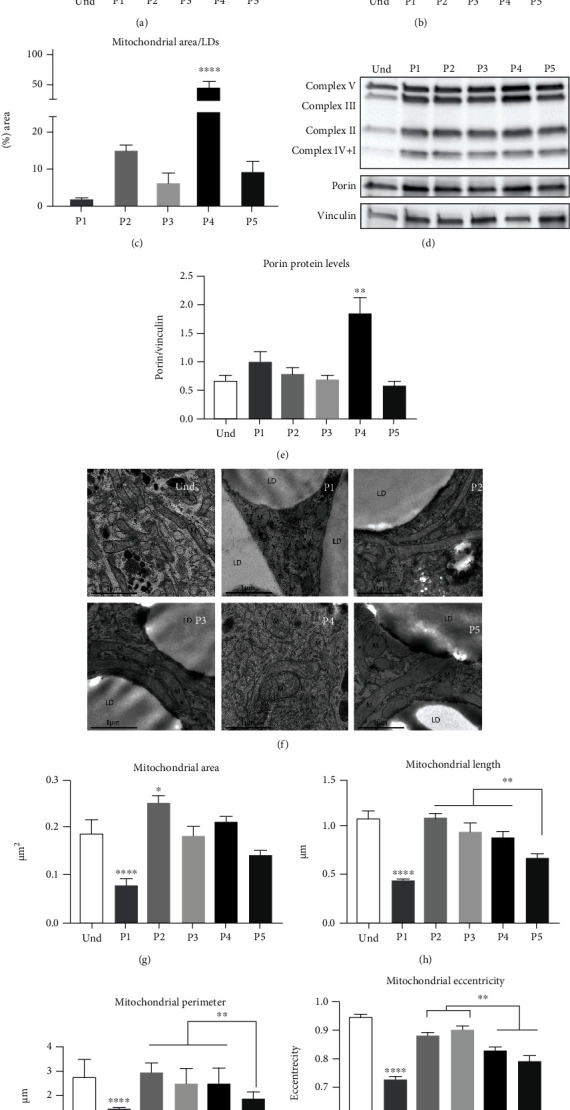
Effect of the differentiation protocols on mitochondrial mass and morphology. Human adipose-derived stem cells were differentiated for 21 days with five adipogenic differentiation protocols. (a) Relative mtDNA amount calculated as a ratio of the mitochondrial gene MT-CYTB to nuclear genes APP and B2M. Quantification of transmission electron microscopy (TEM) images: (b) number of mitochondrial profiles per image and (c) ratio of the mitochondrial area to lipid droplet area quantified with the Microscopy Image Browser (MIB). (d) Protein levels of vinculin, porin, and OXPHOS complex subunits NDUFB8 (CI), SDHB (CII), cytochrome c oxidase subunit 2 COXII (CIV), and ATP5A (CV) were analyzed by western blotting. (e) Quantification of porin level relative to vinculin. Statistical analysis was performed using one-way ANOVA followed by Tukey's post hoc analysis (*n* = 6): ^∗^*p* < 0.05, ^∗∗^*p* < 0.01, ^∗∗∗∗^*p* < 0.0001. Error bars are shown as SEM. (f) TEM images of mitochondria in undifferentiated hASCs and adipocytes differentiated with five protocols. Cells were imaged at 6000x magnification using a JEOL JEM-1400 electron microscope. Quantification of (g) mitochondrial area, (h) length, (i) perimeter, and (j) eccentricity with MIB. hASC3 was used for TEM analysis. At least 500 mitochondria were analyzed for each condition. Statistical analysis was performed using one-way ANOVA followed by Tukey's post hoc analysis: ^∗^*p* < 0.05, ^∗∗^*p* < 0.01, and ^∗∗∗∗^*p* < 0.0001. Error bars are shown as SEM. The statistical differences in (b), (c), (e), and (g) indicate a comparison with all the other protocols.

Since the increased abundance of mitochondria is a hallmark of browning [35, 38], we next analyzed mitochondrial biogenesis using mtDNA amount as a readout. The amount of mtDNA was highest in P1 and P4 adipocytes (Figure 5(a)). We also quantified mitochondrial number in TEM images, which were significantly increased with P4 (Figure 5(b)). Additionally, we measured the ratio of the mitochondrial area to lipid droplet area per cell. Consistent with our previous findings, this ratio was remarkably increased in P4, indicating the abundance of mitochondria over lipid content, the signature of brown-like adipocytes [29, 59] (Figure 5(c)). Using immunoblotting, we then evaluated mitochondrial mass based on mitochondrial outer membrane protein porin expression, which was significantly elevated with P4 compared with the other protocols (Figures 5(d) and 5(e)). Additionally, we analyzed protein levels of OXPHOS complex subunits (Figure 5(d)) and observed that these were significantly increased with P4 compared with other protocols (Supplementary Figure 2(a)–(d). However, when protein levels were normalized per mitochondrion using the mitochondrial outer membrane protein porin as a loading control, the effect of the P4 protocol on OXPHOS complex subunits was lost (Supplementary Figure 2(e)–(h)). This suggests increased mitochondrial content in P4 but without an enrichment of OXPHOS complexes per mitochondrion.

To evaluate mitochondrial morphology, we analyzed the area, length, perimeter, and eccentricity of mitochondria profiles using TEM (Figure 5(f)). Mitochondrial morphology is a distinctive feature between brown and white adipocytes, as the mitochondrial network is mainly elongated (fusion prevailed) in white adipocytes, whereas the network in brown adipocytes is fragmented (fission prevailed) [27, 38]. Mitochondrial fission is crucial for brown adipocyte uncoupling and thermogenesis, while fusion is associated with more efficient ATP production [60]. We observed that P2- and P3-differentiated cells possess the most elongated mitochondria, which was evidenced by increased area, length, perimeter, and eccentricity (Figures 5(g)–5(j)). Eccentricity is the parameter of an object varying between 1 (object is a line) and 0 (object is a sphere). We observed decreased mitochondrial eccentricity in P4 and especially in P1 and P5, suggesting a fragmentation of the mitochondrial network, a hallmark of browning (Figure 5(j)). The relatively small size of mitochondria in P1 and P5 could explain the enrichment of complex IV+I subunits observed in these protocols after normalization of protein levels to porin (Supplementary Figure 2(e)). Taken together, these results suggest that adipocytes differentiated with the INDO-based P4 protocol exhibit a brown adipocyte-like phenotype with an increased number of fragmented mitochondria. Additionally, mitochondrial fragmentation was detected in P1 (Rosi+T3) and P5 (commercial) protocols together. Instead, P2- and P3-differentiated adipocytes (Rosi) exhibited a white adipocyte mitochondrial phenotype.

### 3.6. The Mechanism Mediating the Effects on Mitochondrial Biogenesis

**Figure 6:**
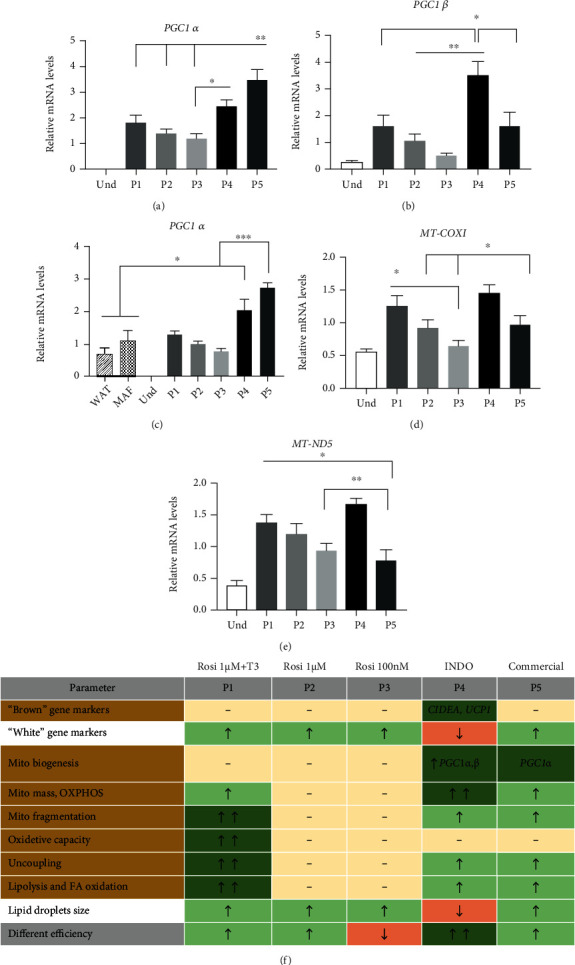
Expression levels of mitochondrial biogenesis and mtDNA-encoded genes. Human adipose-derived stem cells were differentiated for 21 days with five adipogenic differentiation protocols. Relative mRNA expression of nuclear-encoded genes (a) *PGC1α* and (b) *PGC1β* measured by RT-qPCR. (c) Relative mRNA expression of *PGC1α* in *in vitro*-differentiated adipocytes, white adipose tissue (WAT), and mature adipocyte fraction (MAF) of the same subjects measured by RT-qPCR. Relative mRNA expression of mitochondrial-encoded genes (d) *MT-COXI* and (e) *MT-ND5* measured by RT-qPCR. Statistical analysis was performed using one-way ANOVA (*n* = 6) followed by Tukey's post hoc analysis: ^∗^*p* < 0.05, ^∗∗^*p* < 0.01, and ^∗∗∗^*p* < 0.001. Error bars are shown as SEM. (f) Summary table indicating adipocyte and mitochondrial parameters in hASCs differentiated for 21 days with five adipogenic differentiation protocols.

To study the mechanism driving the observed changes in mitochondrial biogenesis and dynamics, we analyzed levels of the key mitochondrial biogenesis regulators *PGC1α* and *PGC1β*. The expression level of *PGC1α* was significantly higher with protocols P4 and P5, but *PGC1β* was higher only in P4 compared to other conditions (Figures 6(a) and 6(b)). To confirm our results with *ex vivo* samples, we compared the expression levels of *PGC1α* in *in vitro*-differentiated adipocytes to *ex vivo* subcutaneous WAT and MAF. Our findings confirm that both P4 (INDO) and P5 (commercial) have higher expression levels of *PGC1α* than what is naturally occurring in *ex vivo* WAT and MAF (Figure 6(c)). Given that part of the mitochondrial proteins is encoded in the mitochondrial genome, we assessed the expression levels of two mtDNA-encoded transcripts, *MT-ND5* and *MT-COXI*, which are downstream targets of PGC1*α*. We observed elevated expression levels of *MT-COXI* and *MT-ND5* in P4 and P1 (Figures 6(d) and 6(e)). Taken together, our data indicate that increased mitochondrial biogenesis and its downstream effects on *MT*- gene expression with P4 (INDO) could result from the induction of PGC1*α* or PGC1*β*.

## 4. Discussion

In recent decades, mitochondria have been recognized as important regulators of white adipose tissue metabolic health, and mitochondrial dysfunction has been associated with obesity and related metabolic complications [7, 9, 61]. The availability and relative ease of adipogenic differentiation of hASCs make them an attractive tool to elucidate the mechanisms behind mitochondrial dysfunction in obesity and to study compounds to reactivate dysfunctional mitochondria. However, a marked limitation of white adipocytes in *in vitro* studies is the lack of a standardized differentiation protocol [34–40]. Despite the large number of hASC studies, a detailed characterization of the effects that differentiation methods have on the white adipocyte phenotype has not been performed before. In this study, we assessed the metabolic and mitochondrial phenotype of adipocytes differentiated with five widely used differentiation protocols. We propose that the best method for white adipocyte differentiation that does not induce browning of white adipocytes is the differentiation protocol that contains Rosi 1 *μ*M (P2).

Adipogenic differentiation is a complex process that requires serial activation of transcription factors, among which the most crucial are peroxisome proliferator-activated receptor gamma (PPAR*γ*) and the CCAAT/enhancer-binding protein (C/EBP) groups of genes [62, 63]. Insulin was discovered among the first adipogenic differentiation media compounds to induce spontaneous lipid accumulation in ASCs *in vitro* by activating phosphatidylinositol-3 kinase, AKT1/2, and mammalian targets of rapamycin pathways [60]. Furthermore, addition of dexamethasone and IBMX improves differentiation by stimulating the glucocorticoid receptor and cAMP signaling pathways, respectively [60, 64, 65]. These are standard compounds of adipogenic differentiation media (Supplementary Table 1) and were part of the tested differentiation protocols in this study.

Adipogenic differentiation *in vitro* is typically improved by the addition of the nonsteroidal anti-inflammatory drug INDO or thiazolidinediones such as Rosi. Both activate PPAR*γ*, the master regulator of adipogenesis. INDO has been widely used for adipogenic differentiation of precursors from both white and brown fat depots [60, 66–71]. Typically, cells are exposed to INDO during the entire differentiation period, or the compound is used cyclically (Supplementary Table 1). Rosi is also an insulin sensitizer, which makes it one of the main compounds of adipogenic differentiation media. The use of Rosi during differentiation is highly variable among protocols (Supplementary Table 1). Another common compound is the thyroid hormone T3, which improves adipogenesis by binding to thyroid hormone receptor A [60]. T3 is regularly used in combination with Rosi and occasionally with INDO. Although T3 is generally applied only for the induction period, in some cases, it is used for both induction and maintenance (Supplementary Table 1). In this study, we investigated the effect of PPAR*γ* activators and T3 on the adipocyte metabolic and mitochondrial phenotype.

We compared differentiation protocols that include the PPAR*γ* agonist Rosi (P2, P3) or an unspecified PPAR*γ* agonist in commercial media (P5) during 7 days of induction. Compared with other adipogenic compounds, the effect of Rosi on the adipocyte and mitochondrial phenotype is the most studied [40, 72]. Rosi promotes white-to-brown adipocyte transdifferentiation in both rodents and humans by activation of mitochondrial biogenesis and function, FAO, and thermogenesis via UCP1 [34–38, 40]. However, the incubation period of Rosi appears to be crucial to induce browning. Greater than 9-day exposure to Rosi induces white-to-brown adipocyte conversion with abundant thermogenically active mitochondria [37, 55, 73]. We compared the effect of Rosi concentrations of 1 *μ*M (P2) and 100 nM (P3) to identify the minimal dose to induce differentiation but prevent the browning effect. Induction for 7 days with 1 *μ*M (P2) or 100 nM (P3) of Rosi did not affect gene expression of any browning markers or lipid metabolism. In both protocols, mitochondria possessed mainly an elongated network and the coupling efficiency of mitochondrial respiration was significantly higher compared to any other differentiation condition. However, the differentiation efficiency with 100 nM Rosi (P3) was low, which limits its use *in vitro*. Our results indicate that Rosi successfully differentiated primary hASCs into white adipocytes, but it should be used with caution in differentiation media to prevent white-to-brown conversion or reduction of differentiation efficiency. Thus, based on efficient differentiation and lipid accumulation, a moderate increase in mitochondrial mass and respiration, and white adipocyte gene expression profile, we propose P2 (Rosi 1 *μ*M) as the most optimal protocol for white adipocyte differentiation *in vitro* (Figure 5(e)).

The effect of INDO on mitochondria has not been extensively investigated. However, a recent study revealed a dose-dependent browning effect on 3T3-L1 cells and human primary brown and subcutaneous white adipocytes [74]. In contrast, Wang et al. reported that INDO decreased *UCP1* expression in cultures of human brown adipocytes [75]. In this study, the INDO-based protocol (P4) showed the highest differentiation efficiency. However, we observed increased expression of *UCP1*, *UCP2*, and *CIDEA*, together with a significant decrease on *LEPTIN*, which is a typical signature of *in vitro* browning of white adipocytes [40, 55, 73, 76]. Furthermore, we observed increased mitochondrial biogenesis and mass, mitochondrial network fragmentation, and reduced lipid droplet size, all of which are brown adipocyte features (Figure 5(e)). Thus, our data indicate that exposure to INDO during the whole differentiation period can lead to browning of white adipocytes, which might be dose dependent [74]. Traditionally, INDO is used in combination with IBMX and a high concentration of insulin to enhance differentiation [68] (Supplementary Table 1). Therefore, further studies are required to fully understand the effect of INDO for white adipocyte differentiation.

T3 increases basal metabolic rate and energy expenditure in brown adipocytes by direct stimulation of UCP1, UCP2, and lipolysis gene expression in brown adipocytes, human subcutaneous WAT, mature white adipocytes, and *in vitro*-differentiated subcutaneous adipocytes [77–80]. T3 used in white adipocyte differentiation media was shown to promote mitochondrial biogenesis via PGC1*α* and PGC1*β*, mtDNA replication and transcription, and oxygen consumption [39]. In line with Lee et al. [39], this study indicates that the T3-containing differentiation protocol (P1) resulted in increased mtDNA amount,mitochondrial respiration, lipolysis rate, and FAO-related gene expression. Moreover, we also observed that T3 induced a large fragmentation of the mitochondrial network and shifted respiration towards uncoupling (Figure 5(e)). A fragmented mitochondrial network is a brown adipocyte feature [27] and is crucial for thermogenesis and uncoupling [81–87]. As no such effect was detected in P2 containing the same compounds as P1 (except T3), this study suggests that T3 induces browning markers *in vitro*. However, unlike in the classical brown phenotype, thyroid hormones also increase lipid accumulation [88, 89], which was indicated in our study given that P1-differentiated cells formed the largest lipid droplets. Indeed, this could explain why T3 is commonly used for the differentiation of white adipocytes, likely neglecting its impact on mitochondrial parameters.

Lastly, we analyzed a commonly used commercial medium (P5) containing the same compounds as P2 and P3, such as Rosi, DEXA, and IBMX, although the exact concentrations and the type of PPAR*γ* agonist are unknown. We observed that 7-day incubation with commercial medium strongly increased PGC1*α* expression, lipolysis rate, and mitochondrial fragmentation (Figure 5(e)). This could be explained by a possible high concentration of the PPAR*γ* agonist, the type of PPAR agonist used, or both. Overall, this protocol resulted in brown adipocyte-like features as T3 (P1) and INDO (P4) and therefore should be used with caution for *in vitro* differentiation of white adipocytes.

## 5. Conclusions

The lack of a uniform adipogenic protocol is a generally accepted limitation for the *in vitro* utilization of hASCs. In this study, we compared several protocols by setting up a workflow to extensively characterize adipocytes, their phenotype, functionality, and mitochondria. Our data revealed that commonly used differentiation protocols containing INDO, T3, and commercial media induced browning features *in vitro* and consequently should be used with caution when aimed at obtaining a pure culture of white adipocytes. Instead, we propose induction with 1 *μ*M Rosi for 7 days (P2) as a standard differentiation protocol of white adipocytes to minimize the browning effect. Our findings will improve future research in obesity, specifically regarding the role of mitochondria in obesity development and potential treatments. Furthermore, our proposed protocol is beneficial for the study of other adipose tissue pathologies, such as lipodystrophies, and for basic adipocyte biology research.

## Figures and Tables

**Table 1:** Human white adipocyte differentiation protocols compared in this study.

Protocol source		P1		P2		P3		P4		P5	
		Lee et al., 2013 modified 1		Lee et al., 2013 modified 2		Lee et al., 2013 modified 3		Zhang et al., 2013 modified		ZenBio DM2 (commercial)	
Total duration induction time		21 days 7 days		21 days 7 days		21 days 7 days		21 days 21 days		21 days 7 days	
		Induction	Maintenance	Induction	Maintenance	Induction	Maintenance	Induction	Maintenance	Induction	Maintenance
Basic conditions	Medium	DMEM/F12	DMEM/F12	DMEM/F12	DMEM/F12	DMEM/F12	DMEM/F12	DMEM/F12	DMEM/F12	DMEM/F12	DMEM/F12
	Serum	HS 3%	HS 3%	HS 3%	HS 3%	HS 3%	HS 3%	HS 3%		FBS	FBS
Basic compounds	Insulin	100 nM	100 nM	100 nM	100 nM	100 nM	100 nM	850 nM		Unknown	Unknown
	Dexamethasone	1 *μ*M	1 *μ*M	1 *μ*M	1 *μ*M	1 *μ*M	1 *μ*M	1 *μ*M		Unknown	Unknown
	IBMX	0.5 mM		0.5 mM		0.5 mM		0.5 mM		Unknown	Unknown
	Rosiglitazone	1 *μ*M		1 *μ*M		100 nM				Unknown	Unknown
	T3	2 nM									
	Transferrin	10 *μ*g/ml									
	Indomethacin							125 *μ*M			
Additional compounds	Biotin	33 *μ*M	33 *μ*M	33 *μ*M	33 *μ*M	33 *μ*M	33 *μ*M	33 *μ*M		Unknown	Unknown
	Pantothenate	17 *μ*M	17 *μ*M	17 *μ*M	17 *μ*M	17 *μ*M	17 *μ*M	17 *μ*M		Unknown	Unknown

DMEM/F12 = Dulbecco's modified Eagle's medium/Nutrient Mixture F-12; HS = human serum; FBS = fetal bovine serum; IBMX =3-isobutyl-1-methylxanthine; T3 = triiodothyronine.

**Table 2:** White and brown adipocyte features.

Parameter	White	Brown
Brown fat markers	−	+
White fat markers	+	−
Mitochondrial biogenesis	+	++
Mitochondrial mass	+	++
Mitochondrial fragmentation	−	+
Mitochondrial oxidative capacity	+	++
Uncoupling and thermogenesis	−	++
Lipolysis and *β*-oxidation	+	++
Lipid droplet number	+	++
Lipid droplet size	+++	+

## Data Availability

Data may be available from the corresponding author upon reasonable request.

## Supplementary Materials

Supplementary Materials Supplementary Table 1: conditions used for induction of white adipocyte differentiation in publications. Supplementary Table 2: primer sequences for human genes used in this study. Supplementary Table 3: Harmony 4.9 image analysis pipeline. Supplementary Figure 1: fluorescent lipid droplet staining with Bodipy green and quantification of lipid droplet parameters with Harmony software. Supplementary Figure 2: quantification of the OXPHOS protein levels relative to vinculin and porin. Supplementary Figure 1: (a) human adipose-derived stem cells were differentiated for 21 days with five adipogenic differentiation protocols. Lipid droplets were stained with Bodipy green and the nucleus with Hoechst 33342, and cells were imaged at 5x magnification with a PerkinElmer Opera Phenix confocal microscope. All six cell lines are represented. Quantification of (b) lipid droplet area per cell and (c) lipid droplet mean size with Harmony software. Lipid droplets were stained with Bodipy green and imaged at 5x magnification with a PerkinElmer Opera Phenix confocal microscope. Statistical analysis was performed using one-way ANOVA (*n* = 6) followed by Tukey's post hoc analysis: ^∗^*p* < 0.05; ^∗∗^*p* < 0.01. Error bars are shown as SEM. Supplementary Figure 2: quantification of the (a) CIV+I, (b) CII, (c) CIII, and (d) CV protein levels relative to vinculin. Quantification of the (e) CIV+I, (f) CII, (g) CIII, and (h) CV protein levels relative to porin. Statistical analysis was performed using one-way ANOVA (*n* = 6) followed by Tukey's post hoc analysis: ^∗^*p* < 0.05. Error bars are shown as SEM. The statistical differences in (c) and (d) indicate a comparison with all the other protocols.
