# Supplementary material for: Preventing White Adipocyte Browning during Differentiation In Vitro: The Effect of Differentiation Protocols on Metabolic and Mitochondrial Phenotypes
Source: Stem Cells Int. 2022 Apr 5;2022:3308194. doi: 10.1155/2022/3308194 (PMC9005291; doi:10.1155/2022/3308194)
Supplement: Supplementary Materials — Supplementary Table 1: conditions used for induction of white adipocyte differentiation in publications. Supplementary Table 2: primer sequences for human genes used in this study. Supplementary Table 3: Harmony 4.9 image analysis pipeline. Supplementary Figure 1: fluorescent lipid droplet staining with Bodipy green and quantification of lipid droplet parameters with Harmony software. Supplementary Figure 2: quantification of the OXPHOS protein levels relative to vinculin and porin. Supplementary Figure 1: (a) human adipose-derived stem cells were differentiated for 21 days with five adipogenic differentiation protocols. Lipid droplets were stained with Bodipy green and the nucleus with Hoechst 33342, and cells were imaged at 5x magnification with a PerkinElmer Opera Phenix confocal microscope. All six cell lines are represented. Quantification of (b) lipid droplet area per cell and (c) lipid droplet mean size with Harmony software. Lipid droplets were stained with Bodipy green and imaged at 5x magnification with a PerkinElmer Opera Phenix confocal microscope. Statistical analysis was performed using one-way ANOVA (n = 6) followed by Tukey's post hoc analysis: ∗p < 0.05; ∗∗p < 0.01. Error bars are shown as SEM. Supplementary Figure 2: quantification of the (a) CIV+I, (b) CII, (c) CIII, and (d) CV protein levels relative to vinculin. Quantification of the (e) CIV+I, (f) CII, (g) CIII, and (h) CV protein levels relative to porin. Statistical analysis was performed using one-way ANOVA (n = 6) followed by Tukey's post hoc analysis: ∗p < 0.05. Error bars are shown as SEM. The statistical differences in (c) and (d) indicate a comparison with all the other protocols. [file 3308194.f1.zip › Supplementary tables/Table 1 supp.docx]

| **Publication** | **Compound, concentration** | | | | | | | | | | | | | |
| --- | --- | --- | --- | --- | --- | --- | --- | --- | --- | --- | --- | --- | --- | --- |
|  | **Pantothenate** | **Biotin** | **Insulin** | **DEXA** | **IBMX** | **PPARγ agonists** | | | | **INDO** | **T3** | **Transferrin** | **HC** | **Induction days** |
|  |  |  |  |  |  | **Rosiglitazone** | **Troglitazone** | **Pioglitazone** | **Ciglitazone** |  |  |  |  |  |
| **Hyväri et al., 2018** | 17 μM | 33 μM | 100 nM | 1 μM | 0.25 mM |  |  |  |  |  |  |  |  | 3-7 |
| **Baker et al., 2017** | 17 μM | 33 μM | 500 nM | 1 μM | 0.5 mM |  |  |  | 1 μM |  | 2 nM | 10 μg/ml |  | 16-20 |
| **Brännmark et al., 2014** | 17 μM | 33 μM | 100 nM | 1 μM | 0.5 mM |  |  | 1 μM |  |  |  |  |  | 7 |
| **Harms et al., 2019** | 17 μM | 33 μM | 100 nM | 1 μM | 0.5 mM |  |  | 1 μM |  |  |  |  |  | 7 |
| **Hemmrich et al., 2004** |  |  | 66 nM | 100 nM | 0.5 mM |  |  | 300 nM |  |  | 1 nM | 10 μg/ml |  | 7 |
| **Jia et al., 2012** |  |  | 85 nM | 1 μM | 0.5 mM | 500 nM |  |  |  |  | 2 nM | 10 μg/ml |  | 3-7 |
| **Keuper et al., 2014** |  |  | 20 nM | 25 nM | 0.25 mM | 2 μM |  |  |  |  | 2 nM | 10 μg/ml | 100 nM | 7 |
| **Kristof et al., 2015** | 17 μM | 33 μM | 20 nM | 25 nM | 0.5 mM | 2 μM |  |  |  |  | 2 nM | 10 μg/ml | 100 nM | 21 |
| **Lee et al., 2013** | 17 μM | 33 μM | 100 nM | 100 nM | 0.5 mM | 1 μM |  |  |  |  | 2 nM | 10 μg/ml |  | ? |
| **Lequeux et al., 2009** |  |  | 0,15 UI/ml | 500 nM | 0.5 mM | 1 μM |  |  |  |  | 2 nM |  | 10 nM | 3-4 |
| **Ninomiya et al., 2010** |  |  | 5 μg/ml | 250 nM | 0.5 mM | 1 μM |  |  |  |  |  |  |  | 3+6 |
| **Pachon-Pena et al., 2011** |  |  | 100 nM | 1 μM | 0.5 mM | 5 μM |  |  |  |  |  |  |  | 14 |
| **Pisani et al., 2011** |  |  | 85 nM | 1 μM | 0.5 mM | 100 nM |  |  |  |  | 2 nM |  |  | 25 |
| **Skurk et al., 2007** |  |  | 66 nM | 100 nM | 0.5 mM | 2 μM |  |  |  |  | 1 nM | 10 μg/ml | 100 nM | 4 |
| **Todorcevic et al., 2017** | 17 μM | 33 μM | 100 nM | 1 μM | 0.25 mM |  | 4 μM |  |  |  | 10 nM | 10 μg/ml | 100 nM | 4 |
| **Drehmer et al., 2017** |  |  | 10 μg/ml | 1 μM | 0.5 mM |  |  |  |  | 200 μM |  |  |  | 4*3 |
| **Huang et al., 2017** |  |  | 10 μg/ml | 100 nM | 0.5 mM |  |  |  |  | 200 μM |  |  |  | 3-5 |
| **Kakudo et al., 2017** |  |  | 10 μg/ml | 1 μM | 0.5 mM |  |  |  |  | 200 μM |  |  |  | ? |
| **Karlsson et al., 2009** |  |  | 5 μg/ml | 1 μM | 0.5 mM |  |  |  |  | 60 μM |  |  |  | 21 |
| **Lee et al., 2016** |  |  | 10 μg/ml | 1 μM | 0.5 mM |  |  |  |  | 200 μM |  |  |  | 4*3 |
| **Liu et al., 2012** |  |  | 10 μg/ml | 1 μM | 0.5 mM |  |  |  |  | 100 μM |  |  |  | 21 |
| **Mauney et al., 2005** |  |  | ? | 500 nM | 0.5 mM |  |  |  |  | 50 μM |  |  |  | 21 |
| **Sekiya et al., 2004** |  |  | ? | 500 nM | 0.5 mM |  |  |  |  | 50 μM |  |  |  | 21 |
| **Zhang et al., 2013** |  |  | 5 μg/ml | 1 μM | 0.5 mM |  |  |  |  | 50 μM |  |  |  | 7-21 |
| **Bellemare et al., 2009** | **Zen-Bio DM2** | | | | | | | | | | | | | 3 |
| **Bujalska et al., 2008** | **Zen-Bio DM2** | | | | | | | | | | | | | 3-9 |
| **Carter et al., 2013** | **Zen-Bio DM2** | | | | | | | | | | | | | 3-21 |
| **Liu et al., 2016** | **Zen-Bio DM2** | | | | | | | | | | | | | 21 |
| **Liu et al., 2017** | **Zen-Bio DM2** | | | | | | | | | | | | | 3+ |
| **Ortega et al., 2009** | **Zen-Bio DM2** | | | | | | | | | | | | | 12 |
| **Satish et al., 2015** | **Zen-Bio DM2** | | | | | | | | | | | | | 4 |
| **Tajiri et al., 2014** | **Zen-Bio DM2** | | | | | | | | | | | | | 21 |

Supplementary Table 1: Conditions used for induction of white adipocyte differentiation in publications.
