# Supplementary material for: Preventing White Adipocyte Browning during Differentiation In Vitro: The Effect of Differentiation Protocols on Metabolic and Mitochondrial Phenotypes
Source: Stem Cells Int. 2022 Apr 5;2022:3308194. doi: 10.1155/2022/3308194 (PMC9005291; doi:10.1155/2022/3308194)
Supplement: Supplementary Materials — Supplementary Table 1: conditions used for induction of white adipocyte differentiation in publications. Supplementary Table 2: primer sequences for human genes used in this study. Supplementary Table 3: Harmony 4.9 image analysis pipeline. Supplementary Figure 1: fluorescent lipid droplet staining with Bodipy green and quantification of lipid droplet parameters with Harmony software. Supplementary Figure 2: quantification of the OXPHOS protein levels relative to vinculin and porin. Supplementary Figure 1: (a) human adipose-derived stem cells were differentiated for 21 days with five adipogenic differentiation protocols. Lipid droplets were stained with Bodipy green and the nucleus with Hoechst 33342, and cells were imaged at 5x magnification with a PerkinElmer Opera Phenix confocal microscope. All six cell lines are represented. Quantification of (b) lipid droplet area per cell and (c) lipid droplet mean size with Harmony software. Lipid droplets were stained with Bodipy green and imaged at 5x magnification with a PerkinElmer Opera Phenix confocal microscope. Statistical analysis was performed using one-way ANOVA (n = 6) followed by Tukey's post hoc analysis: ∗p < 0.05; ∗∗p < 0.01. Error bars are shown as SEM. Supplementary Figure 2: quantification of the (a) CIV+I, (b) CII, (c) CIII, and (d) CV protein levels relative to vinculin. Quantification of the (e) CIV+I, (f) CII, (g) CIII, and (h) CV protein levels relative to porin. Statistical analysis was performed using one-way ANOVA (n = 6) followed by Tukey's post hoc analysis: ∗p < 0.05. Error bars are shown as SEM. The statistical differences in (c) and (d) indicate a comparison with all the other protocols. [file 3308194.f1.zip › Supplementary tables/Table 2 supp.docx]

Supplementary Table 2: Primer sequences for human genes used in this study.

| **Gene** | **Gene name** | **Forward (5’ - 3’)** | **Reverse (5’ - 3’)** |
| --- | --- | --- | --- |
| **IPO8** | Importin 8 | AAGAAACCGCGCTTGAGGGG | ATCCTCGCTGAGTGGTGCCA |
| **GUSB** | Glucuronidase Beta | GTCTGCGGCATTTTGTCGG | CACACGATGGCATAGGAATGG |
| **APP** | Amyloid Beta Precursor Protein | TGTGTGCTCTCCCAGGTCTA | CAGTTCTGGATGGTCACTGG |
| **B2M** | Beta-2-Microglobulin | TGCTGTCTCCATGTTTGATGTATCT | TCTCTGCTCCCCACCTCTAAGT |
| **ADIPOQ** | Adiponectin | TGGTGAGAAGGGTGAGAA | AGATCTTGGTAAAGCGAATG |
| **FABP4** | Fatty Acid Binding Protein 4 | ACTGGGCCAGGAATTTGAGG | CTCGTGGAAGTGACGCCTT |
| **LEP** | Leptin | GCTGTGCCCATCCAAAAAGTCC | CCCAGGAATGAAGTCCAAACCG |
| **CIDEA** | Cell Death Inducing DFFA Like Effector A | TTATGGGATCACAGACTAAG | TGCTCCTGTCATGGTTGGAGA |
| **PPARγ** | Peroxisome Proliferator Activated Receptor Gamma | TACTGTCGGTTTCAGAAATGAC | GTCAGCGGACTCTGGATTCAG |
| **PGC1α** | PPARγ Coactivator 1 Alpha | AGCCTCTTTGCCCAGATCTT | GGCAATCCGTCTTCATCCAC |
| **PGC1β** | PPARγ Coactivator 1 Beta | CCACATCCTACCCAACATCAAG | CACAAGGCCGTTGACTTTTAGA |
| **UCP1** | Uncoupling protein 1 | CAAATCAGCTCCGCCTCTCT | AATGAATACTGCCACTCCTCCAG |
| **UCP2** | Uncoupling protein 2 | CCCTCCTGAAAGCCAACCTC | AGAAGCCTGCCCCAAAGG |
| **MT-ND5** | Mitochondrially Encoded NADH: Ubiquinone Oxidoreductase Core Subunit 5 | AGGCGCTATCACCACTCTGTTCG | AACCTGTGAGGAAAGGTATTCCT |
| **MT-COX1** | Mitochondrially Encoded Cytochrome C Oxidase I | GGATGCATACACCACATGAA | AGCGAAGGCTTCTCAAATCA |
| **MT-CYTB** | Mitochondrially Encoded Cytochrome B | GCCTGCCTGATCCTCCAAAT | AAGGTAGCGGATGATTCAGCC |
| **LIPE** | Lipase E, Hormone Sensitive Type | TCAGTGTCTAGGTCAGACTGG | AGGCTTCTGTTGGGTATTGGA |
| **ACADM** | Acyl-CoA Dehydrogenase Medium Chain | CGGGGTTCGGGCGATG | TCCTGGTTCACGTTGTCGAT |
| **ACADS** | Acyl-CoA Dehydrogenase Small Chain | TTGCAGCCCAGGTGGATAAG | GTAGGCCAGGTAATCGAGGC |
| **ZIC1** | Zic Family Member 1 | CACGCGGGACTTTCTGTTC | TGCCCGTTGACCACGTTAG |
| **TBX1** | T-Box Transcription Factor 1 | CTACGACCACTATCTCGGGG | TGGGGCAATAGTCGTAGGAG |
| **TMEM26** | Transmembrane Protein 26 | ATGGAGGGACTGGTCTTCCTT | CTTCACCTCGGTCACTCGC |
