# Supplementary material for: Preventing White Adipocyte Browning during Differentiation In Vitro: The Effect of Differentiation Protocols on Metabolic and Mitochondrial Phenotypes
Source: Stem Cells Int. 2022 Apr 5;2022:3308194. doi: 10.1155/2022/3308194 (PMC9005291; doi:10.1155/2022/3308194)
Supplement: Supplementary Materials — Supplementary Table 1: conditions used for induction of white adipocyte differentiation in publications. Supplementary Table 2: primer sequences for human genes used in this study. Supplementary Table 3: Harmony 4.9 image analysis pipeline. Supplementary Figure 1: fluorescent lipid droplet staining with Bodipy green and quantification of lipid droplet parameters with Harmony software. Supplementary Figure 2: quantification of the OXPHOS protein levels relative to vinculin and porin. Supplementary Figure 1: (a) human adipose-derived stem cells were differentiated for 21 days with five adipogenic differentiation protocols. Lipid droplets were stained with Bodipy green and the nucleus with Hoechst 33342, and cells were imaged at 5x magnification with a PerkinElmer Opera Phenix confocal microscope. All six cell lines are represented. Quantification of (b) lipid droplet area per cell and (c) lipid droplet mean size with Harmony software. Lipid droplets were stained with Bodipy green and imaged at 5x magnification with a PerkinElmer Opera Phenix confocal microscope. Statistical analysis was performed using one-way ANOVA (n = 6) followed by Tukey's post hoc analysis: ∗p < 0.05; ∗∗p < 0.01. Error bars are shown as SEM. Supplementary Figure 2: quantification of the (a) CIV+I, (b) CII, (c) CIII, and (d) CV protein levels relative to vinculin. Quantification of the (e) CIV+I, (f) CII, (g) CIII, and (h) CV protein levels relative to porin. Statistical analysis was performed using one-way ANOVA (n = 6) followed by Tukey's post hoc analysis: ∗p < 0.05. Error bars are shown as SEM. The statistical differences in (c) and (d) indicate a comparison with all the other protocols. [file 3308194.f1.zip › Supplementary tables/Table 3 supp.docx]

Supplementary table 3: Harmony 4.9 image analysis pipeline.

| **Input Image** | **Input** |  |  |
| --- | --- | --- | --- |
|  | Flatfield Correction : Advanced |  |  |
|  | Stack Processing : Maximum Projection |  |  |
|  | Create Global Image |  |  |
|  | Min. Global Binning : Dynamic |  |  |
|  |  |  |  |
| **Find Nuclei** | **Input** | **Method** | **Output** |
|  | Channel : DAPI (global) | Method : B | Output Population : Nuclei (global) |
|  | ROI : Imaged Area (global) | Common Threshold : 0.5 |  |
|  | ROI Region : Imaged Area | Area : > 30 µm² |  |
|  |  | Splitting Coefficient : 5 |  |
|  |  | Individual Threshold : 0.59 |  |
|  |  | Contrast : > 0 |  |
|  |  |  |  |
| **Calculate Morphology Properties** | **Input** | **Method** | **Output** |
|  | Population : Nuclei (global) | Method : Standard | Property Prefix : Nucleus |
|  | Region : Nucleus | Area |  |
|  |  | Roundness |  |
|  |  |  |  |
| **Select Population** | **Input** | **Method** | **Output** |
|  | Population : Nuclei (global) | Method : Filter by Property | Output Population : Nuclei (global) Selected |
|  |  | Nucleus Area [µm²] : > 40 |  |
|  |  | Nucleus Roundness : > 0.4 |  |
|  |  | Nucleus Area [µm²] : < 900 |  |
|  |  | Boolean Operations : F1 and F2 and F3 |  |
|  |  |  |  |
| **Find Cytoplasm** | **Input** | **Method** | **Output** |
|  | Channel : DAPI (global) | Method : D |  |
|  | Nuclei : Nuclei (global) Selected | Individual Threshold : 0.18 |  |
|  |  | Restrictive Region : Imaged Area |  |
|  |  |  |  |
| **Select Population** | **Input** | **Method** | **Output** |
|  | Population : Nuclei (global) Selected | Method : Filter by Property | Output Population : Cytoplasm selected |
|  |  | Nucleus Area [µm²] : < 20000 |  |
|  |  | Nucleus Area [µm²] : > 40 |  |
|  |  | Boolean Operations : F1 and F2 |  |
|  |  |  |  |
| **Calculate Intensity Properties** | **Input** | **Method** | **Output** |
|  | Channel : Alexa 488 (global) | Method : Standard | Property Prefix : Intensity Cytoplasm Bodipy |
|  | Population : Cytoplasm selected | Mean |  |
|  | Region : Cytoplasm | Standard Deviation |  |
|  |  | Coefficient of Variance |  |
|  |  | Median |  |
|  |  | Sum |  |
|  |  | Maximum |  |
|  |  | Minimum |  |
|  |  | Contrast |  |
|  |  |  |  |
| **Calculate Texture Properties** | **Input** | **Method** | **Output** |
|  | Channel : Alexa 488 (global) | Method : SER Features | Property Prefix : Cell Bodipy |
|  | Population : Cytoplasm selected | Scale : 2 px |  |
|  | Region : Cytoplasm | Normalization by : Kernel |  |
|  |  | SER Spot |  |
|  |  | SER Hole |  |
|  |  | SER Edge |  |
|  |  | SER Ridge |  |
|  |  | SER Valley |  |
|  |  | SER Saddle |  |
|  |  | SER Bright |  |
|  |  | SER Dark |  |
|  |  |  |  |
| **Select Population** | **Input** | **Method** | **Output** |
|  | Population : Cytoplasm selected | Method : Linear Classifier | Output Population A : differentiated |
|  |  | Number of Classes : 2 | Output Population B : non-differentiated |
|  |  | Intensity Cytoplasm Bodipy Mean |  |
|  |  | Intensity Cytoplasm Bodipy StdDev |  |
|  |  | Intensity Cytoplasm Bodipy Median |  |
|  |  | Intensity Cytoplasm Bodipy Maximum |  |
|  |  | Intensity Cytoplasm Bodipy Minimum |  |
|  |  | Intensity Cytoplasm Bodipy Sum |  |
|  |  | Intensity Cytoplasm Bodipy CV [%] |  |
|  |  | Intensity Cytoplasm Bodipy Contrast |  |
|  |  | Cell Bodipy SER Spot 2 px |  |
|  |  | Cell Bodipy SER Hole 2 px |  |
|  |  | Cell Bodipy SER Edge 2 px |  |
|  |  | Cell Bodipy SER Ridge 2 px |  |
|  |  | Cell Bodipy SER Valley 2 px |  |
|  |  | Cell Bodipy SER Saddle 2 px |  |
|  |  | Cell Bodipy SER Bright 2 px |  |
|  |  | Cell Bodipy SER Dark 2 px |  |
|  |  |  |  |
| **Find Spots** | **Input** | **Method** | **Output** |
|  | Channel : Alexa 488 (global) | Method : B | Output Population : Spots nondifferentiated |
|  | ROI : non-differentiated | Detection Sensitivity : 0.2 |  |
|  | ROI Region : Cell | Splitting Sensitivity : 0.5 |  |
|  |  | Calculate Spot Properties |  |
|  |  |  |  |
| **Find Spots** | **Input** | **Method** | **Output** |
|  | Channel : Alexa 488 (global) | Method : B | Output Population : Spots differentiated |
|  | ROI : differentiated | Detection Sensitivity : 0.2 |  |
|  | ROI Region : Cell | Splitting Sensitivity : 0.5 |  |
|  |  | Calculate Spot Properties |  |
|  |  |  |  |
| **Select Population** | **Input** | **Method** | **Output** |
|  | Population : Spots nondifferentiated | Method : Filter by Property | Output Population : Droplets nondifferentiated |
|  |  | Corrected Spot Intensity : > 1000 |  |
|  |  | Spot Area [px²] : > 3 |  |
|  |  | Boolean Operations : F1 and F2 |  |
|  |  |  |  |
| **Select Population** | **Input** | **Method** | **Output** |
|  | Population : Spots differentiated | Method : Filter by Property | Output Population : Droplets differentiated |
|  |  | Corrected Spot Intensity : > 1000 |  |
|  |  | Spot Area [px²] : > 3 |  |
|  |  | Boolean Operations : F1 and F2 |  |
|  |  |  |  |
| **Calculate Morphology Propertie** | **Input** | **Method** | **Output** |
|  | Population : Droplets nondifferentiated | Method : Standard | Property Prefix : Droplets nondifferentiated |
|  | Region : Spot | Area |  |
|  |  | Roundness |  |
|  |  | Width |  |
|  |  | Length |  |
|  |  | Ratio Width to Length |  |
|  |  |  |  |
| **Calculate Morphology Properties** | **Input** | **Method** | **Output** |
|  | Population : Droplets differentiated | Method : Standard | Property Prefix : Droplets differentiated |
|  | Region : Spot | Area |  |
|  |  | Roundness |  |
|  |  | Width |  |
|  |  | Length |  |
|  |  | Ratio Width to Length |  |
|  |  |  |  |
| **Calculate Intensity Properties** | **Input** | **Method** | **Output** |
|  | Channel : Alexa 488 (global) | Method : Standard | Property Prefix : Intensity Droplets nondiff Alexa 488 |
|  | Population : Droplets nondifferentiated | Mean |  |
|  | Region : Spot | Sum |  |
|  |  |  |  |
| **Calculate Intensity Properties** | **Input** | **Method** | **Output** |
|  | Channel : Alexa 488 (global) | Method : Standard | Property Prefix : Intensity Droplets diff Alexa 488 |
|  | Population : Droplets differentiated | Mean |  |
|  | Region : Spot | Sum |  |
|  |  |  |  |
|  |  |  |  |
|  |  |  |  |
| **Calculate Properties** | **Input** | **Method** | **Output** |
|  | Population : non-differentiated | Method : By Related Population | Property Suffix : per Cell |
|  |  | Related Population : Droplets nondifferentiated |  |
|  |  | Number of Droplets nondifferentiated |  |
|  |  | Corrected Spot Intensity |  |
|  |  | Spot Area [px²] |  |
|  |  | Droplets nondifferentiated Area [µm²] |  |
|  |  | Droplets nondifferentiated Roundness |  |
|  |  | Droplets nondifferentiated Width [µm] |  |
|  |  | Droplets nondifferentiated Length [µm] |  |
|  |  | Droplets nondifferentiated Ratio Width to Length |  |
|  |  | Intensity Droplets nondiff Alexa 488 Mean |  |
|  |  | Intensity Droplets nondiff Alexa 488 Sum |  |
|  |  |  |  |
| **Calculate Properties** | **Input** | **Method** | **Output** |
|  | Population : differentiated | Method : By Related Population | Property Suffix : per Cell |
|  |  | Related Population : Droplets differentiated |  |
|  |  | Number of Droplets differentiated |  |
|  |  | Corrected Spot Intensity |  |
|  |  | Spot Area [px²] |  |
|  |  | Droplets differentiated Area [µm²] |  |
|  |  | Droplets differentiated Roundness |  |
|  |  | Droplets differentiated Width [µm] |  |
|  |  | Droplets differentiated Length [µm] |  |
|  |  | Droplets differentiated Ratio Width to Length |  |
|  |  | Intensity Droplets diff Alexa 488 Mean |  |
|  |  | Intensity Droplets diff Alexa 488 Sum |  |
|  |  |  |  |
|  |  |  |  |
| **Define Results** | **Results** |  |  |
|  | Method : List of Outputs |  |  |
|  | Population : Nuclei (global) Selected |  |  |
|  | Number of Objects |  |  |
|  | Apply to All : Mean |  |  |
|  | Nucleus Area [µm²] : Mean |  |  |
|  | Nucleus Roundness : Mean |  |  |
|  | Cytoplasm selected : Mean |  |  |
|  | Population : Nuclei (global) |  |  |
|  |  |  |  |
|  | **Population : Droplets nondifferentiated** |  |  |
|  | Number of Objects |  |  |
|  | Corrected Spot Intensity : Mean |  |  |
|  | Droplets nondifferentiated Area [µm²] : Mean |  |  |
|  | Intensity Droplets nondiff Alexa 488 Mean : Mean |  |  |
|  | Intensity Droplets nondiff Alexa 488 Sum : Sum |  |  |
|  |  |  |  |
|  | **Population : Droplets differentiated** |  |  |
|  | Number of Objects |  |  |
|  | Corrected Spot Intensity : Mean |  |  |
|  | Droplets differentiated Area [µm²] : Mean |  |  |
|  | Intensity Droplets diff Alexa 488 Mean : Mean |  |  |
|  | Intensity Droplets diff Alexa 488 Sum : Sum |  |  |
|  |  |  |  |
|  | **Population : differentiated** |  |  |
|  | Number of Objects |  |  |
|  | Intensity Cytoplasm Bodipy Mean : Mean |  |  |
|  | Intensity Cytoplasm Bodipy Sum : Sum |  |  |
|  | Number of Spots per Area of Cell : Mean |  |  |
|  | Number of Droplets differentiated- per Cell : Mean |  |  |
|  | Droplets differentiated Area [µm²]- Mean per Cell : Mean |  |  |
|  | Intensity Droplets diff Alexa 488 Mean- Mean per Cell : Mean |  |  |
|  | Intensity Droplets diff Alexa 488 Sum- Sum per Cell : Sum |  |  |
|  |  |  |  |
|  | **Population : non-differentiated** |  |  |
|  | Number of Objects |  |  |
|  | Intensity Cytoplasm Bodipy Mean : Mean |  |  |
|  | Intensity Cytoplasm Bodipy Sum : Sum |  |  |
|  | Number of Spots per Area of Cell : Mean |  |  |
|  | Number of Droplets nondifferentiated- per Cell : Mean |  |  |
|  | Droplets nondifferentiated Area [µm²]- Mean per Cell : Mean |  |  |
|  | Intensity Droplets nondiff Alexa 488 Mean- Mean per Cell : Mean |  |  |
|  | Intensity Droplets nondiff Alexa 488 Sum- Sum per Cell : Sum |  |  |
|  |  |  |  |
|  | **Method : Formula Output** |  |  |
|  | Formula : a/b |  |  |
|  | Population Type : Objects |  |  |
|  | Variable a : differentiated - Number of Objects |  |  |
|  | Variable b : Cytoplasm selected - Number of Objects |  |  |
|  | Output Name : Ratio differentiated |  |  |
|  |  |  |  |
|  | **Method : Formula Output** |  |  |
|  | Formula : a/b |  |  |
|  | Population Type : Objects |  |  |
|  | Variable a : non-differentiated - Number of Objects |  |  |
|  | Variable b : Cytoplasm selected - Number of Objects |  |  |
|  | Output Name : Ratio non-differentiated |  |  |
|  |  |  |  |
|  | **Object Results** |  |  |
|  | Population : Nuclei (global) Selected : Use Selected Well Results |  |  |
|  | Population : Droplets nondifferentiated : Use Selected Well Results |  |  |
|  | Population : Droplets differentiated : Use Selected Well Results |  |  |
|  | Population : differentiated : Use Selected Well Results |  |  |
|  | Population : non-differentiated : Use Selected Well Results |  |  |
| Acapella version: 5.0.1.124082. Timestamp: 2021-06-04 16:25:15 +0300. |  |  |  |
